# Supplementary material for: Efficacy of cilastatin sodium in a translational large animal crush syndrome model
Source: Commun Med (Lond). 2026 Mar 12;6:246. doi: 10.1038/s43856-026-01411-1 (PMC13121696; doi:10.1038/s43856-026-01411-1)
Supplement: Supplementary file 2 — Supplementary Information [file 43856_2026_1411_MOESM2_ESM.pdf]

Supplementary Methods

Animals

All animal procedures were approved by the Institutional Animal Care and Use Committee of Oregon Health & Science University, and separately, by the Animal Care and Use Review Office of the US Army Medical Research and Development Command. Animals used for these investigations were Female Yorkshire-Landrace crossbred pigs weighing 40-50 kg; they were housed in a temperature and light-controlled vivarium for 7 days prior to surgery, with food and water provided *ad libitum* until 18h prior to surgery.

Randomization and blinding

Animals were block randomized to four groups (cilastatin, cilastatin+calcitriol, vehicle and no impact) in 2:2:2:1 ratio using the R library *randomizeR*[89]. Personnel performing animal care were blinded to the intervention for the duration of the project and did not perform data analysis. The renal pathologist was blinded to treatment groups.

Cilastatin activity assay

Because of supply chain instability in 2021 and 2022, cilastatin sodium was purchased from two vendors (Toronto Research Chemicals, Toronto CA and MedChemExpress, Monmouth NJ). To determine cilastatin activity, aliquots of the two cilastatin sodium preparations used in the study ("bulk supplier" preparations) and 2 used in our published small animal studies (sml1283 and s5734, Sigma-Aldrich, CA, US, "comparator" preparations) were subjected to a well-characterized renal dipeptidase activity assay[46, 47]. Briefly, the dipeptidase substrate glycyl-D-phenylalanine (MilliporeSigma, Ontario Canada) was added to 1 mM cilastatin sodium or phosphate-

buffered saline (the positive control), pre-incubated with 0.0035 nmol recombinant dipeptidase-1 (DPEP1, MilliporeSigma, Ontario Canada) in reaction assay buffer (50 µL/well). After 40-minute incubation, fluorescence was measured at 414 nm after excitation at 317nm, and the relative activity quantified as fraction of the mean of control fluorescence.

Drug preparation and administration

On the morning of each surgery, cilastatin sodium or vehicle (according to the randomization scheme) was prepared in sterile conditions in a location physically distant from the surgical suite by a technician who was not a member of the surgical team. Immediately after anesthesia induction, the pig was weighed and the weight communicated to the drug preparation technician. Cilastatin sodium was prepared by adding 100 mg/kg of previously-prepared concentrated solution (Cilastatin sodium 400 mg/mL in Plasma-Lyte A) to a 500 mL bag of Plasma-Lyte A (starting composition: sodium 140 mmol, potassium 5 mmol, magnesium 1.5 mmol, chloride 98 mmol, acetate 27 mmol, gluconate 23 mmol in 1 L water). To ensure blinding identical appearance of vehicle and drug was assured by mixing cilastatin sodium into the Plasma-Lyte bag; equal volume of Plasma-Lyte A being withdrawn. The drug (or vehicle) bag was then covered with a brown translucent polyethylene bag and transported to the surgical suite, where all personnel were unaware of the bag's contents. Calcitriol (1 µg) was added to a second, 125 mL bag; vehicle bags contained no calcitriol.

Animal instrumentation and anesthesia

Pigs were fasted for 18 hours prior to surgery to prevent aspiration under general anesthesia. Water was available ad libitum. On the day of the experiment, animals were

sedated with 8mg/kg (tiletamine/zolazepam and 0.005mg/kg glycopyrrolate and placed in dorsal recumbancy on the operating table. Electrocardiography leads and a cutaneous oxygen saturation probe were applied. Induction of anesthesia was performed with 5% Isoflurane and greater than 20% oxygen via face mask. Animals were endotracheally intubated by direct laryngoscopy and mechanically ventilated with maintenance isoflurane at 1-3% throughout surgery.

To ensure a stable airway for the 48-hour experiment, a tracheotomy was performed. Using aseptic technique, an incision was made at the midline of the neck via blunt and sharp dissection to expose the tracheal rings. After temporarily reducing the fraction of inspired oxygen to 20% and deflation of the endotracheal tube balloon, an incision was made between the 3rd and 4th tracheal ring and a 7.0 re-enforced endotracheal tube was advanced to 17 cm at the level of the trachea. The animal remained mechanically ventilated and anesthetized with isoflurane via the tracheostomy tube for the duration of the experiment, and end-tidal carbon dioxide (etCO2), inspired oxygen, expired oxygen, and inspired/expired isoflurane concentrations continuously monitored using an anesthesia gas monitoring system.

The neck was then exposed for placement of a 7 French (Fr) catheter in the carotid artery and a 7 Fr catheter in the internal or external jugular vein. All ports were aspirated and flushed. To provide analgesia, buprenorphine 0.01 mg/kg was administered via the jugular catheter. Arterial pressure was monitored via the carotid catheter. Following the placement of the catheters, baseline blood samples were collected. Metabolic and electrolyte panel (I-STAT; Abbott Labs, Vancouver, WA) was measured to ensure the animals were normal with respect to coagulopathy. The animal received ketamine via

continuous infusion for augmentation of maintenance anesthesia and analgesia at dose of 1-5 mg/kg/hr; this was titrated to ensure the absence of signs of discomfort Lastly, a ventral midline incision was made and a laparotomy performed. A cystostomy was made and a suprapubic balloon-tipped Foley urinary catheter was placed and the laparotomy closed.

Large animal crush syndrome model

Following preparation, anesthesia, and instrumentation, a captive bolt gun (Schermer Stunner Model MKL) was loaded with a 6.8/15 caliber, No. 3 yellow-coded charge containing 200g of propellant (Schermer)[90]. The blunt tip of the captive bolt was placed on the rear hindquarters of the animal, targeting the muscle mass midline to the femur. The tip was further blunted with cardboard padding to spread impact. Two impacts were applied to each hindquarter of the animal (4 total). No impact animals were treated identically but the impacts were not administered. 30 minutes after impact, test substances were administered and animals began to receive continuous IV infusion of 5% dextrose-Lactated Ringers solution at a rate of 10 mL/kg/hr for the first 3 hours, 5 mL/kg/hr for the following 3 hours, and then 2.5 mL/kg/hr for the remainder of the experiment. 5-50 mL of arterial and/or venous blood was withdrawn on a pre-set schedule (see manuscript **Fig. 2**) for autoanalyzer and other plasma measurements (below).

Management of hyperkalemia: If animals developed hyperkalemia (≥5.0 mEq/L), they were treated with 10mL of calcium gluconate, 10 units of regular insulin, and a bolus of 50g of dextrose, repeated as necessary every hour until the potassium level was <5.0 mEq/L (“hyperkalemia intervention”). Plasma potassium levels were checked using the

autoanalyzer (iStat) both 1 hour after hyperkalemia intervention and 3 hours after hyperkalemia intervention.

Management of hypotension: In the event of sustained (>=20 minutes) mean arterial pressure of <40mmHg a 10mL/kg lactated Ringers bolus was administered over 30 minutes. This was repeated as necessary if mean arterial pressure was not above 40mmHg two hours after administration. These criteria were empirically determined during model development to avoid excessive mortality or excessive fluid administration.

Endpoint and euthanasia: 48h after drug administration, anesthesia was deepened, the laparotomy was re-opened and immediately following ligation of the renal artery and vein, a nephrectomy was performed for pathological examination; block nephrectomy samples were placed in formalin. Immediately after nephrectomy, the animal was euthanized by lethal overdose of intravenous pentobarbital. Death was confirmed by the presence of isoelectric electrocardiographic signal and lack of end-tidal CO_2.

Exclusions: Animals were excluded if they had very abnormal baseline plasma measurements (prior to surgery, **Supplementary Table 1**: Exclusions for baseline abnormalities) or if they failed to develop muscle injury (**Supplementary Table 2**: Exclusions for failure to develop muscle injury). To determine abnormal plasma measurements before surgery, baseline plasma measurements were scaled (converted to z scores) and animals were excluded for absolute value scaled scores >2 (i.e., 2 standard deviations greater or less than the mean). These pre-defined exclusion criteria were applied for baseline plasma measurements which indicated pre-existing abnormalities of the target physiologic systems (namely, renal function, musculoskeletal

injury, and cardiovascular function). The predefined measures were mean arterial pressure (map), weight-based plasma creatine kinase (CK), plasma myoglobin (MB), plasma creatinine (CR), and plasma potassium (K).

Animals which received impact but failed to demonstrate objective evidence of muscle injury were also excluded. This pre-defined exclusion was necessary to account for the possibility of variation in the delivery of explosive-driven impact to the thigh from a hand-held captive bolt device. The injury threshold was determined as the minimum weight-indexed change in plasma creatine kinase from baseline to 6h after impact which resulted in elevated plasma creatinine. Animals which did not meet this threshold were excluded.

<u>Blood and urine sample collection</u>

In addition to the baseline draw after catheter placement, blood samples were withdrawn from the arterial catheter at 1h before impact, immediately before test substance administration (time zero), and 2, 6, 12, 18, 24, and 48h after test substance administration. Samples were collected into lithium heparin or sodium citrate-precoated tubes, quickly centrifuged (1500xg at room temperature for 10 min) to separate plasma. Samples were quickly frozen, the cryoprecipitate was removed, and plasma then stored at -80°C prior to further analysis.

Urine was collected and treated with protease inhibitors immediately before drug administration and at 2, 6, 12, 18, 24, and 48h after drug administration. Urine samples were diluted 1:5 in 100mM sodium phosphate buffer, centrifuged, aliquoted, and stored at -80°C prior to further analysis.

<u>Measurements performed on blood and urine samples</u>

Creatine kinase assay:

Creatine kinase was measured in plasma samples by the OHSU clinical laboratory using a BeckmanCoulter AU analyzer.

Urine and plasma myoglobin assay:

Urine and plasma myoglobin were by ELISA using a commercially-available kit (MYO-9, Life Diagnostics, Wes Chester, PA) following manufacturer instructions. 48h excretion of myoglobin was computed by numerical integration of the hourly values using Simpson's rule. Fractional excretion of myoglobin was computed using simultaneously measurede plasma and urine myoglobin and creatinine values according to the formula:

$$FEMb = 100\ x\ \frac{(U_{Mb}\ xP_{Cr}\ )}{(P_{Mb}\ x\ U_{Cr})}$$

Where "U" denotes a urine value, "P" denotes a plasma value, "Mb" is myoglobin concentration, and "Cr" is creatinine concentration.

Urine porphyrin assay:

This method was adapted for urine from the method of Morrison[48]. Prediluted urine samples were added to a 10-fold volume excess of oxalic acid and heated to 100˚C for 30 min. Fluorescence was then measured at 640nm after excitation at 405nm. A standard curve was prepared using identically-treated prediluted aliquots of purified horse skeletal muscle myoglobin (M0630, Sigma-Aldrich, CA, US) and results reported in mg of porphyrin. 48h excretion of porphyrin was computed by numerical integration of the hourly values using Simpson's rule.

Blood chemistry auto-analysis:

Whole blood samples were analysed using an iStat (Abbott, Orlando FL) autoanalyzer to determine sodium, potassium, chloride, bicarbonate, creatinine, urea nitrogen,

ionized calcium, prothrombin time (Compact Max, Diagnostica Stago, Parsippany, NJ, USA), pH, total carbon dioxide, partial pressure of carbon dioxide, partial pressure of oxygen, oxygen saturation, and base excess.

Determination of acute and direct off-target effects of cilastatin

To determine acute effects of cilastatin on non-renal targets, systemic physiology was assessed during the 12 hours after administration. This time limit was chosen to represent a conservative estimate of the maximal plasma residence time for cilastatin sodium, as the plasma half-time in estimated as 45-60 minutes[49, 50] and 12h accounts for >5 half times even in the presence of >50% GFR loss.

Measurement of urine output:

Urine was collected from the urinary catheter by gravity in a volumetric device. Volume was recorded every hour and reset to zero. for the following hour.

Measurement of glomerular filtration rate (GFR)

Animals received 5 mL intravenous iohexol (OmniPaque 300) at 1 hours, 19 hours, and 43 hours to measure glomerular filtration rate (GFR). Blood samples (~2 mL each) were collected at specific time points following iohexol administration (15, 30, 90, 120, 180, 240, and 300 minutes after iohexol injection). Liquid chromatography/mass spectrometry/mass spectrometry (LC/MS/MS) was used to determine the iohexol concentration in each sample.

Water and methanol (HPLC grade) were obtained from Burdick and Jackson (Muskegon, MI), Perchloric acid were obtained from J.T. Baker brand (Phillipsburg, NJ), 0.22µ spin filters were obtained from Pall (Port Washington, NY). Iohexol and d5 Iohexol internal standard were obtained from Cayman Chemical (Ann Arbor, MI). Citrate pig

plasma was obtained from investigator. Sample vials were from Fisher Scientific (Rockwood, TN). LUNA polar omega column 150 x 2.1 mm was from Phenomenex (Torrance, CA).

Iohexol and d5 iohexol solutions were prepared in methanol at 10 mg/ml and 1 mg/ml, respectively. Iohexol calibrators were prepared in pig citrate plasma by spiking stock solutions and serially diluting for a 6-point curve at 5, 10, 50, 100, 500 and 1000 µg/ml. An initial validation consisted of preparing a series of standards in triplicate, the LLOQ was set to 5 ug/ml with S/N of >10 and variability of <10% and accuracy of +/- 15%. Internal standard was prepared in 5% perchloric acid sample precipitation solution at 1ug/150 µl.

Unknown samples and calibrators were thawed at room temperature. After mixing 10 µl was transferred to 1.5 ml Eppendorf tubes and 150 µl of 5% perchloric acid with internal standard was added. Samples were vigorously vortexed for 3 minutes followed by centrifugation at 17,000 RCF for 5 minutes. 100 µl of supernatant was transferred to 0.22µ spin filters which were subsequently spun at 17,000 RCF for 5 minutes and the cleared filtrate was transferred to sample vials and placed in a 15˚ C autosampler.

The HPLC system was a Shimadzu (Columbia, MD) SIL-20AC XR auto-sampler at 15°C, a CBM-20A system controller, two LC-20AD XR LC pumps, a DGU-20 A5 in-line solvent degasser and a CTO-20AC column oven. The chromatographic method was derived from (1). In brief, Iohexol and d5-Iohexol were resolved on a Phenomenex LUNA Polar Omega C18 column (150x2.1 mm, 3µ, 100 Å) and a Javelin pre-filter at 40°C using an 8 min gradient elution after a 5 µl injection. The solvents were A: 10 mM ammonium acetate in water and B: 10 mM ammonium acetate in methanol. Starting

conditions were 5% B held for 0.5 minutes, ramped to 95% B by 4.1 min, held for 1 min, returned to start over 0.1 min, and re-equilibrated until 8 min. Solvent was directed towards the detector from 2.5 to 4.5 min. The LC system was interfaced to an Applied Biosystems/MDS SCIEX 4000 QTRAP triple quadrupole hybrid linear ion trap mass spectrometer (Foster City, CA) and was used in triple quadrupole mode with multiple reaction monitoring (MRM). It was equipped with a TurboIon ESI source operated in the positive mode with the following settings: source voltage 5.0 kV, nebulizer gas (GS1) 30 psi, heater gas (GS2) 50 psi, curtain gas (CUR) 10 psi, source temperature (TEM) 650 °C and collision associated dissociate gas (CAD) -2. Gases were 99.999% nitrogen. The MRM transitions monitored for quantification were m/z 821.9 ® 803.9.0 for iohexol and m/z 826.9 ® 808.8 for d5-iohexol. Optimal parameters for the two MRM transitions were (iohexol, d5-iohexol): collision energy (CE, V): 31, 31; declustering potential (DP, V) 91,96; collision cell exit potential (CXP, V): 14,14; exit potential (EP, V) was 10 for both compounds. Dwell times were 100 ms and Q1 and Q3 were operated at unit resolution. Instrument control and data acquisition were done with Analyst® (version 1.6.2) and quantification with Multiquant® (version 3.0).

Concentration-time data was then fit to a 2-compartment model using the R package *stats*. If the fit for the 2-compartment model was poor, a 1-compartment model was fit. GFR was calculated as (iohexol dose)/(area under the fitted curve)[51-55].

<u>Pathologic assessment of kidney injury</u>

Sections of nephrectomy blocks were cut, formalin-fixed, and paraffin-embedded, then cut into 6μm slices, mounted, and stained with hematoxylin and eosin. In treatment-blinded fashion, sections were qualitatively analyzed. For quantitative assessment of

tubular damage score[14], effacement of tubular epithelium, epithelial vacuolization, and epithelial necrosis were scored from 0-3 with 3 conveying severe injury. 16 high-power fields were assessed per animal, 8 in the cortex and 8 in the outer medulla. Then, for each animal, the mean score for each parameter was computed for each high-power field, and the weighted tubular damage score was calculated as TDS=(effacement score)+(vacuolization score)+2*(necrosis score). The TDS reported for each animal is the mean of the TDS for all 16 high-power fields for that animal.

Assessment of nonrecovery from AKI.

Clinical thresholds for nonrecovery which increased risk of dialysis requirement or development of chronic kidney disease were identified as 1)failure to recover to the baseline creatinine[59] and 2)failure to recover to 75% of normal GFR. Because in this study GFR was only assessed after anesthesia induction and injury, normal GFR was derived from reported iohexol clearance in similar-weight swine of the same strain[91].

Clinical trial simulation and power analysis

To determine power for a future clinical trial we performed Monte Carlo simulation using the categorical variable of recovery to 70% of the maximal creatinine by 48h as the outcome. First, we identified a relevant human population with similar elevation of creatinine kinase to determine mean and standard deviation of baseline creatinine[92]. Using this data and proportional creatinine change in our study we stochastically modeled maximal and 48h creatinine in simulated study participants in Monte Carlo fashion[93]. Simulated studies were repeated 500 times each with a range of experimental n from 20 to 200. P value and experimental power were then computed for each study and the n required to achieve 80 and 90% power, respectively, reported.

Statistical analysis

Statistical analysis was performed using the R statistical language (R Statistical Computing project) (R Statistical Computing project, packages *stats, lmer, robustlmm*[94]*, emmeans, survival* and *survminer*). All statistical analyses performed and necessary to replicate the results are documented in the shared code (See “Data and code availability”). Descriptive statistics (mean, standard deviation, and standard scores) were used to characterize raw data. Comparisons between groups were conducted using mixed models linear regression and residuals evaluated for concordance with the assumptions of distribution using residual vs. predicted value and quantile plots. Except as noted, results are presented in text and figures as mean ± standard error of the mean (SEM). In several cases, outliers resulted in violation of assumptions of distribution; in these cases robust linear mixed models were instead employed and results are presented as median (25th percentile, 75th percentile). Statistical analyses employed are documented in figure captions. Because of seasonal and sequence-based variation in baseline variables (see **Supplemental Analysis, “Seasonal Variation”**), model terms included the sequential animal ID as a random factor. A preplanned analysis was performed to seek divergence between the cilastatin and cilastatin+calcitriol groups (see **Supplemental Analysis, “Cilastatin vs. Cilastatin+Calcitriol”**). Because these groups demonstrated no difference in pre- or post-drug physiologic measurements, and no difference in outcome, their results were pooled for analysis. Adjustment for multiplicity of groups and repeated measurements was performed using the Šidák correction (R package *emmeans*). Analysis of survival and time-to-intervention was performed using Kaplan-Meier analysis and log-rank tests

of significance (R packages *survival* and *survminer*). Analysis of categorical outcomes including for power analysis was performed with X^2 (R package *stats*).

Supplementary Results

Calcitriol did not cause significant alteration to renal physiology or injury

The study was planned to test vehicle versus cilastatin sodium. In a preplanned analysis, we compared whether renal or other physiology was affected by calcitriol administration in cilastatin-alone and cilastatin+calcitriol-treated animals. Data are presented in **Supplementary Figure 7**, and extended tables documenting statistical comparisons in Supplementary Data 2-12.

Administration of calcitriol did not significantly alter plasma creatine kinase (**Supplementary Figure 7A**, Supplementary Data 2). Next, we investigated effects of calcitriol+cilastatin versus cilastatin alone on heart rate, mean arterial pressure, urine output, and temperature. 6h after drug administration, heart rate was transiently lower in calcitriol-treated animals (**Supplementary Figure 7B**, Supplementary Data 3). The difference in means was ~10% of the mean heart rate, however mean arterial pressure was not changed by calcitriol at this time or any other (**Supplementary Figure 7C**, Supplementary Data 4). Urine output was statistically different at 1, 41, and 42h (**Supplementary Figure 7D** Supplementary Data 5). The mean difference in urine output at 1h was 0.02 mL/kg or less at these time points.

Temperature was not influenced by calcitriol administration. (**Supplementary Figure 7E** Supplementary Data 6). Calcitriol had no effect on creatinine (**Supplementary Figure 7F** Supplementary Data 7) or plasma urea nitrogen (**not shown,** Supplementary Data 8). Administered calcitriol had no effect on 6, 24, or 48h GFR (**Supplementary Figure**

7G Supplementary Data 9) Calcitriol administration did not affect plasma potassium (**Supplementary Figure 7H** Supplementary Data 10). Administered calcitriol did not alter plasma calcium (**Supplementary Figure 7I** Supplementary Data 11). Finally, we tested, in a subset of animals, whether plasma calcitriol was increased by administered calcitriol. There was wide variation, but no statistically significant effect of calcitriol administration on calcitriol plasma level (**Supplementary Figure 7J** Supplementary Data 12). Taken together, these data indicate no effect of plasma calcitriol on renal physiology or outcomes and physiologically nonsignificant effects of calcitriol on heart rate and urine output.

<u>Seasonal variation in baseline physiologic indicators</u>

There was significant correlation between seasonally determined day length and several baseline physiologic values. The correlation between the injury marker, creatine kinase (CK), and day length was the strongest and most significant (**Supplementary Table 3** and **Supplementary Figure 8A**). To determine the temporality of this relationship we plotted time on the x axis with daylength and plasma CK on the y axis; a sine function fit well with an approximately 90-degree phase shift between day length and CK, demonstrating that during Winter, baseline CK was higher, and during Summer, baseline CK was lower (**Supplementary Figure 8B**).

These data indicate that baseline CK and other variables including weight varies considerably with seasonal day length indicating appropriateness of model adjustment for day length. Testing of model adjustment for season, day length, or animal sequence number demonstrated that animal sequence number accounted for the most variation from day length with the most parsimonious number of terms.

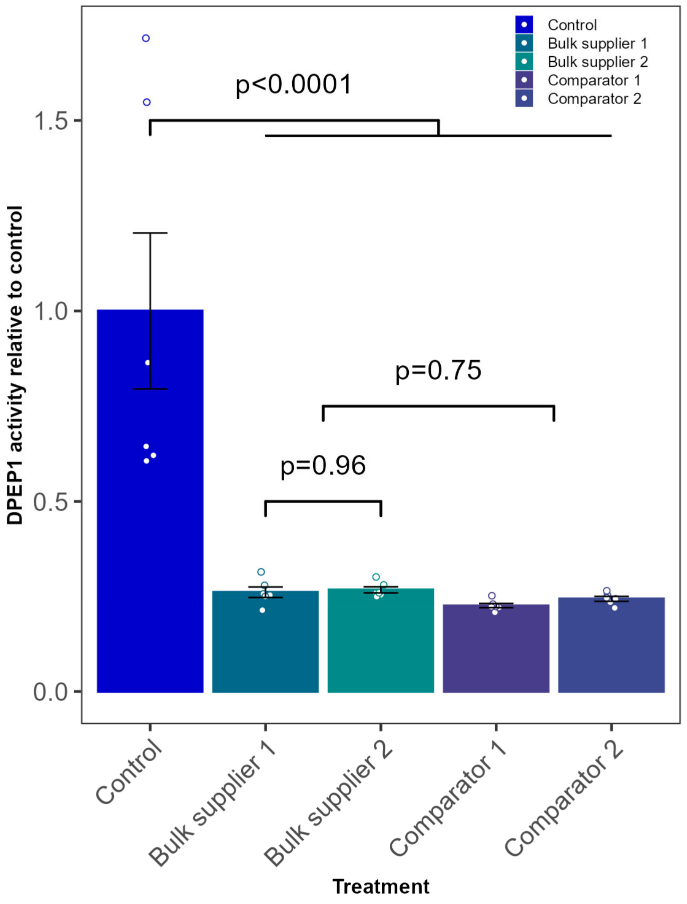

Supplementary figure 1: Renal dipeptidase (DPEP1) activity assay comparing control and cilastatin batches from both suppliers (Bulk suppliers 1 and 2) used in the present study and suppliers used by investigators in prior (rodent) studies (comparators 1 and 2). n=6 replicates for each sample. ANOVA with Sidak post-hoc test. Error bars: mean±SD

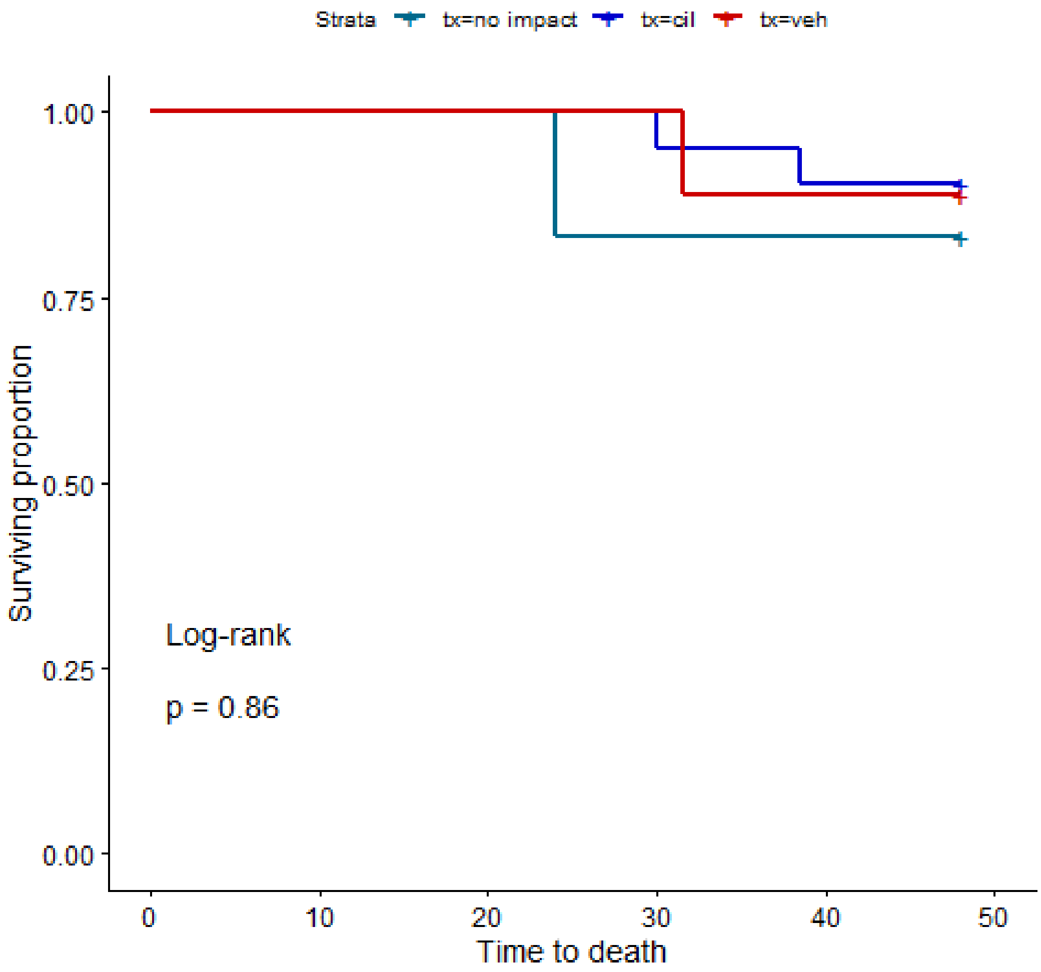

Supplementary figure 2: Survival to the 48h endpoint was not influenced by model or group. n=6 no impact, 9 veh, 21 cil,

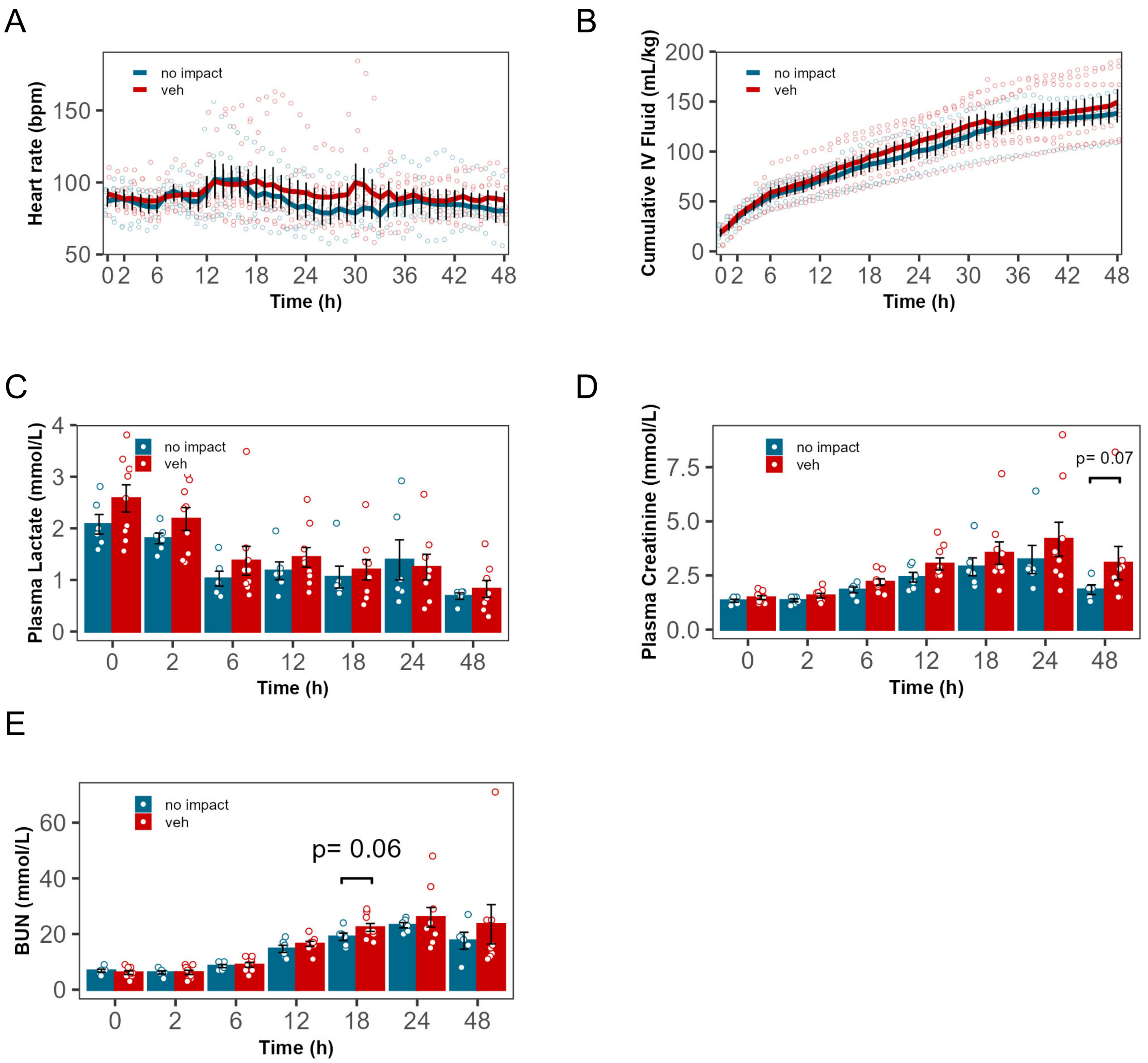

Supplementary figure 3: Heart rate, total fluid intake, plasma lactate, creatinine and plasma urea nitrogen were not significantly changed by thigh impact. N= 6 no impact, 9 veh. A-C: linear mixed models regression. D, E: robust linear mixed models regression.Error bars: A, B, D mean±SEM. C, E: median±IQR.

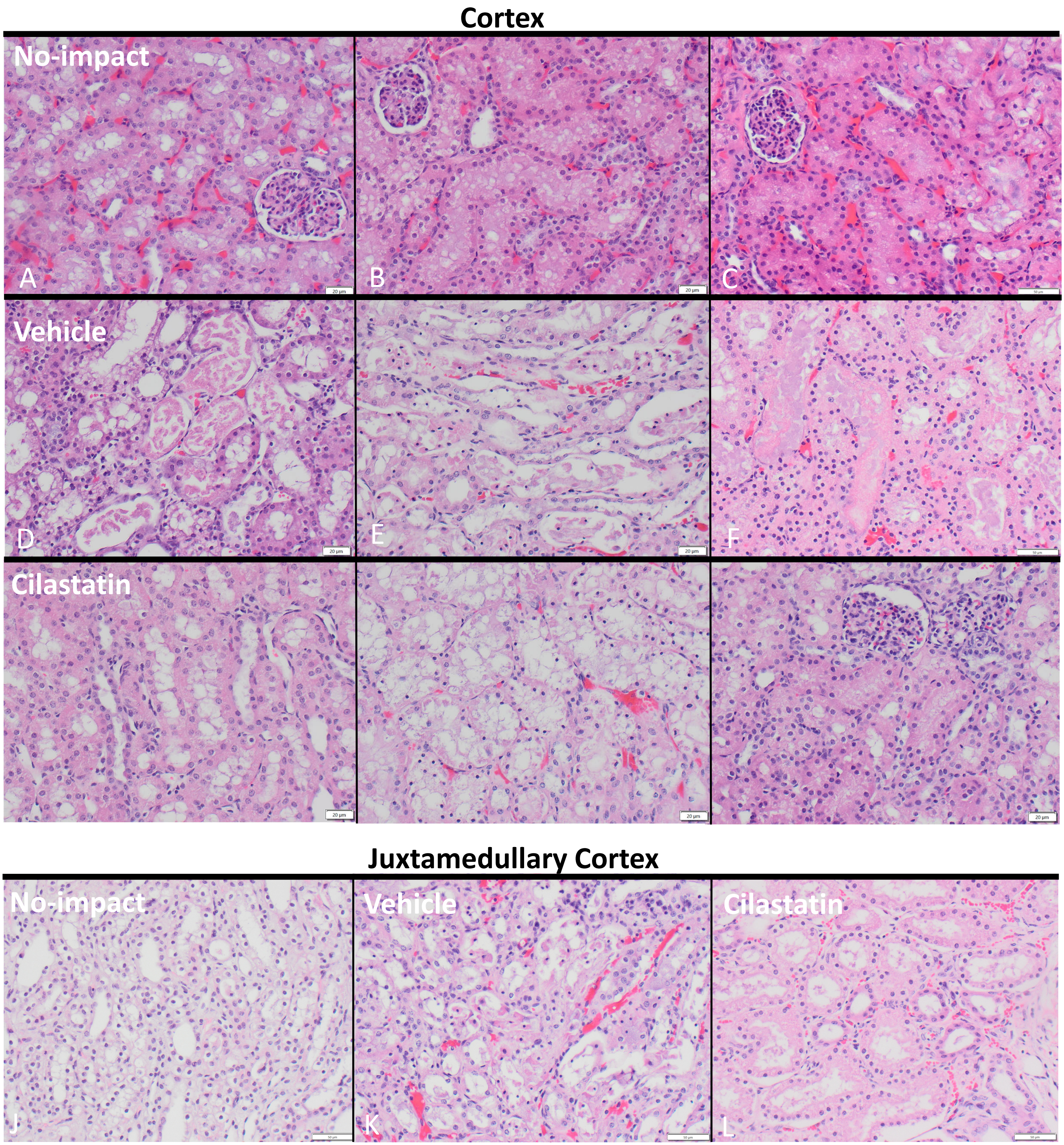

Supplementary Figure 4: Representative renal cortex (top 3 rows) and juxtamedullary cortex and superficial medulla (bottom row) from: A, B, C) Normal histology from 3 separate no-impact animals. D, E, F) Images of acute tubular necrosis and intralumenal casts from 3 animals treated with vehicle only. G, H, I) Variable, mild features of acute tubular injury including attenuation of epithelium (G) and cytoplasmic vacuolization (H) without overt necrosis or casts in 3 animals treated with cilastatin. J, K, L) Juxtamedullar cortex and medulla from J) no-impact animal, normal histology, K) animal treated with vehicle with diffuse tubular necrosis and sloughing of cells into lumen, and L) animal treated with cilastatin, with tubular attenuation and granular cast debris, without overt necrosis. All images H&E, 200x

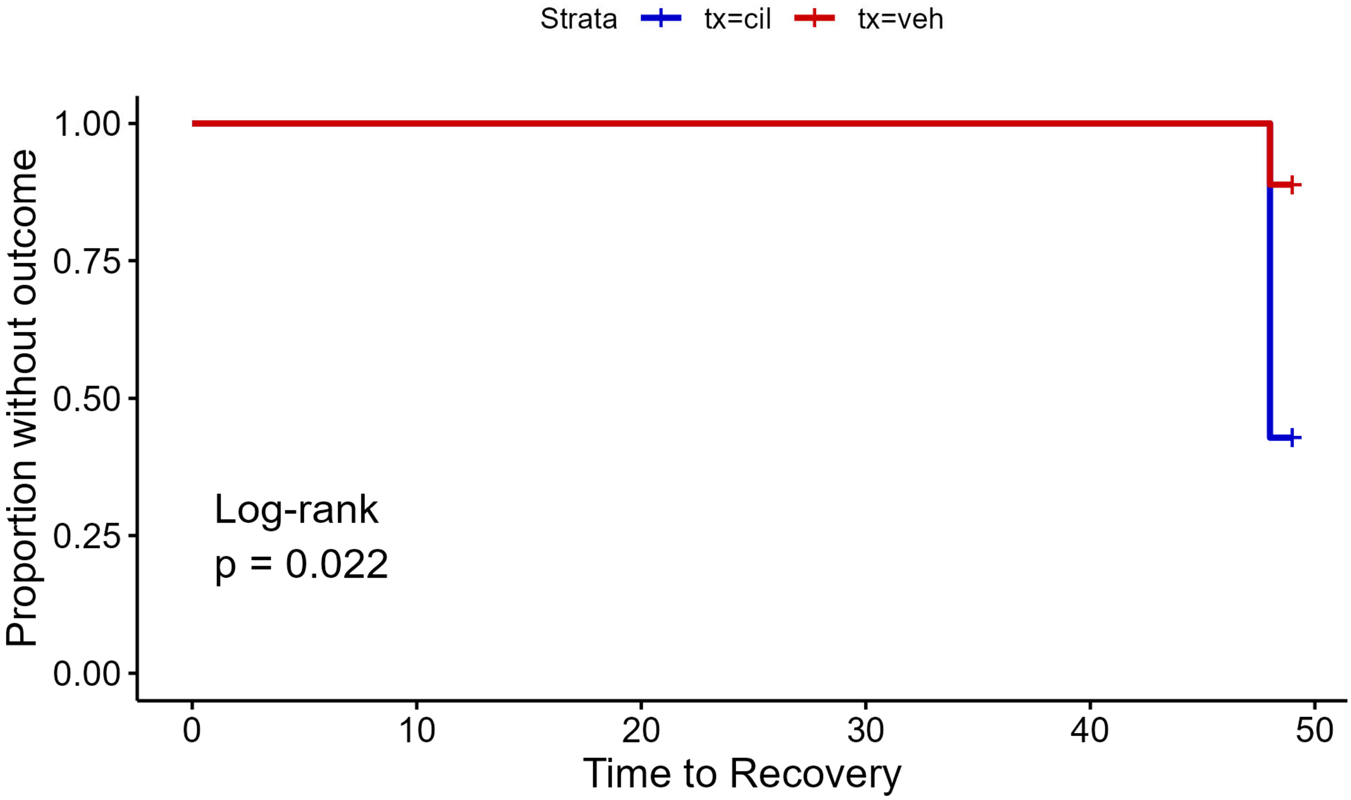

Supplementary figure 5: Time-to-event analysis for recovery of plasma creatinine to <70% of maximal serum creatinine.

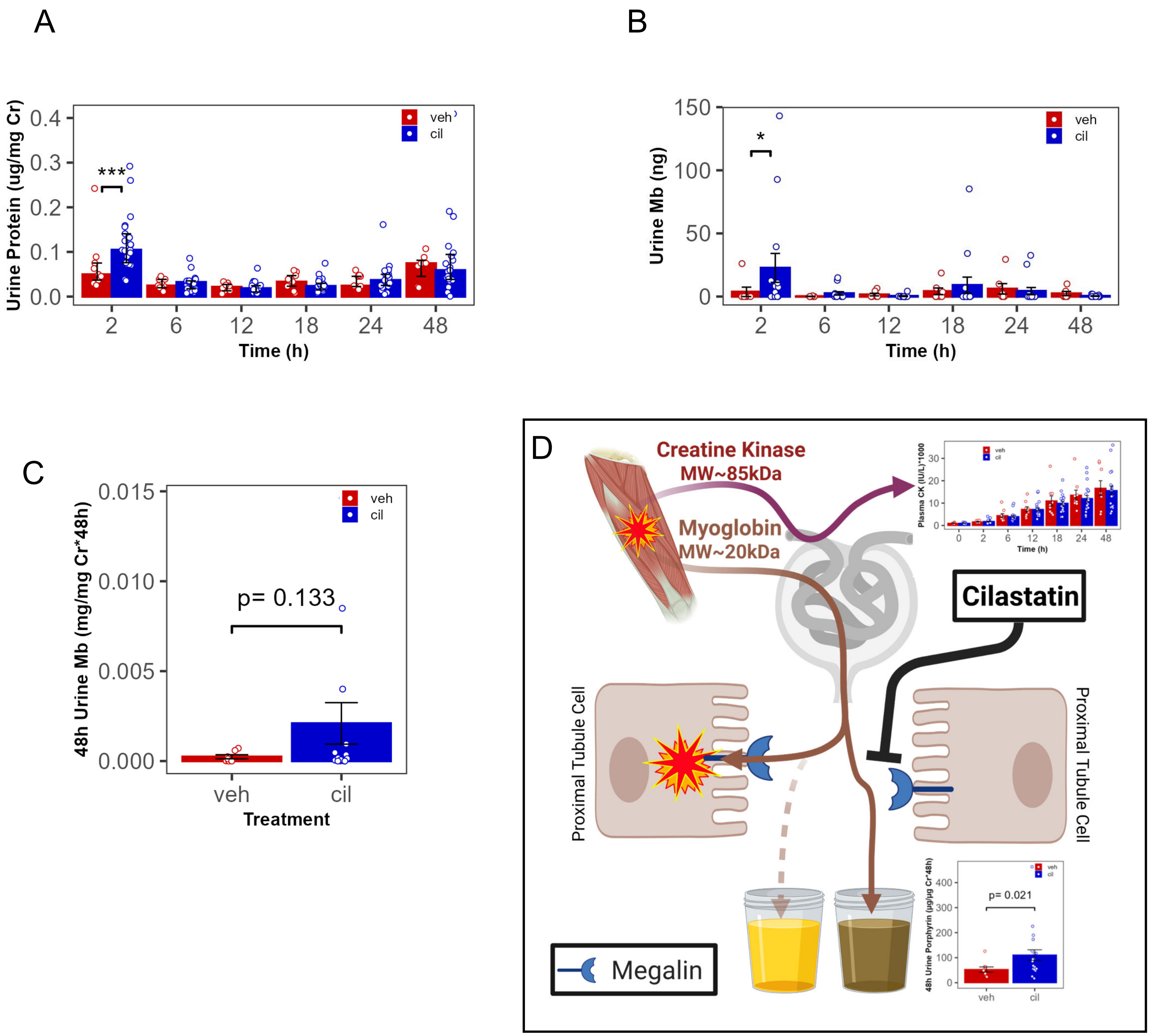

Supplementary figure 6: Urine protein measurements influenced by cilastatin. (A). Urine total protein was increased immediately after cilastatin administration. (B). Myoglobin measurement in urine (unlike in plasma) was insensitive (note many zero values, see text). Nonetheless, myoglobin excretion in the urine was increased 2h after cilastatin administration (C) Over 48h, there was no significant difference in total myoglobin excretion. (D) Cartoon depicts proposed excretory mechanism of cilastatin. Creatine kinase and myoglobin reach the glomerulus after muscle injury. Creatine kinase is too large to filter (molecular weight, MW~85 kilodaltons (kDa), but myoglobin, with MW ~20 kDa filters and reaches the proximal tubule. In vehicle treated animals, most myoglobin binds megalin and enters proximal tubule cells, to which it is toxic. In cil-treated animals, megalin-binding is inhibited, and myoglobin remains in the urine, where it is excreted and can be measured in the urine as porphyrins. A-C: n=9 veh, 21 cil. A, B: robust linear mixed models regression. C: Welch t-test. Error bars: A,B: median±IQR, C mean ±SEM.

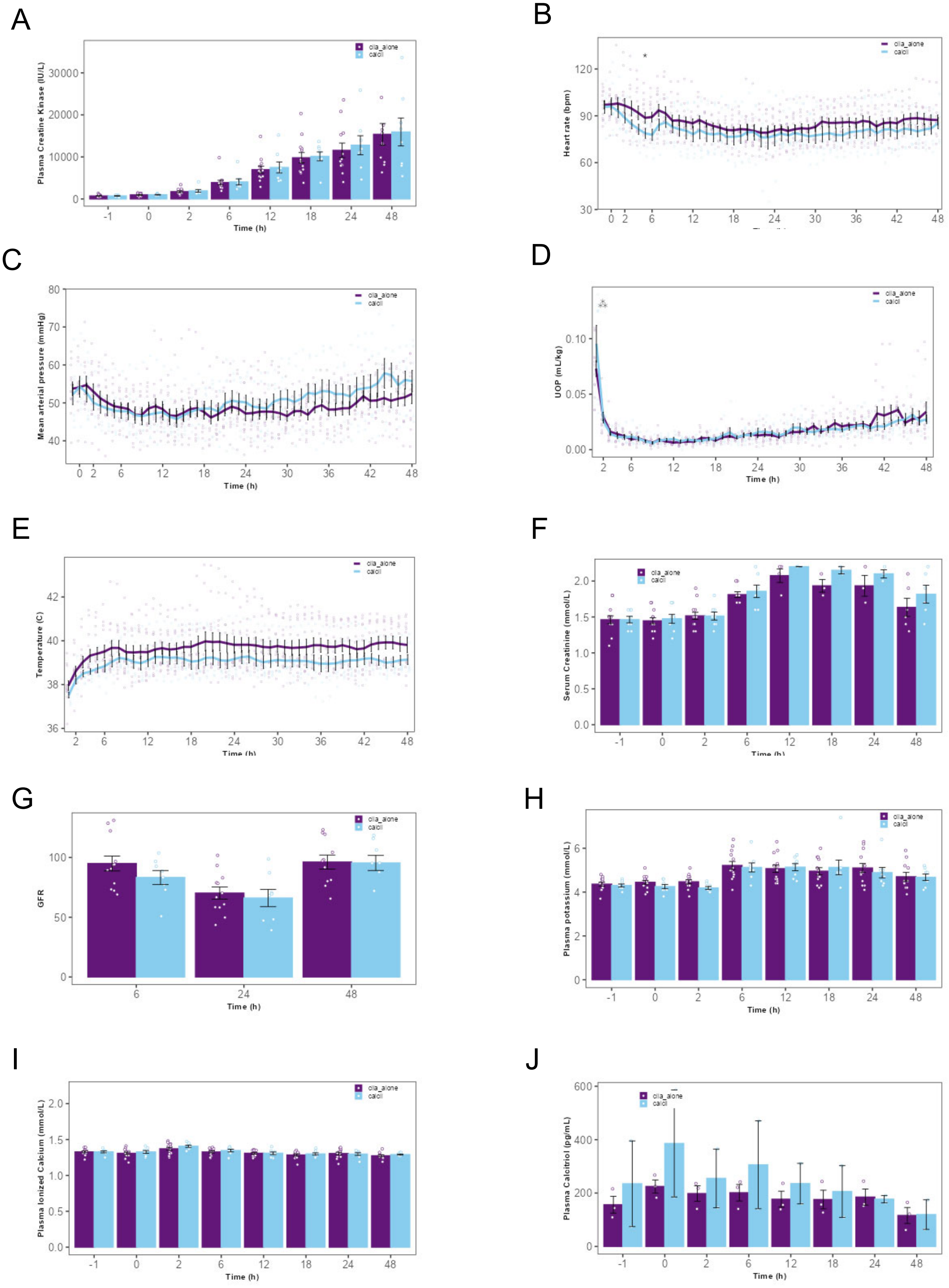

Supplementary figure 7: Calcitriol administration did not significantly change physiologic status or kidney function with the exception of physiologically insignificant reduction in heart rate (~10% of mean heart rate for 1 h) and increase in urine output (<=0.02 mL/kg/h for 3 discontinuous h).

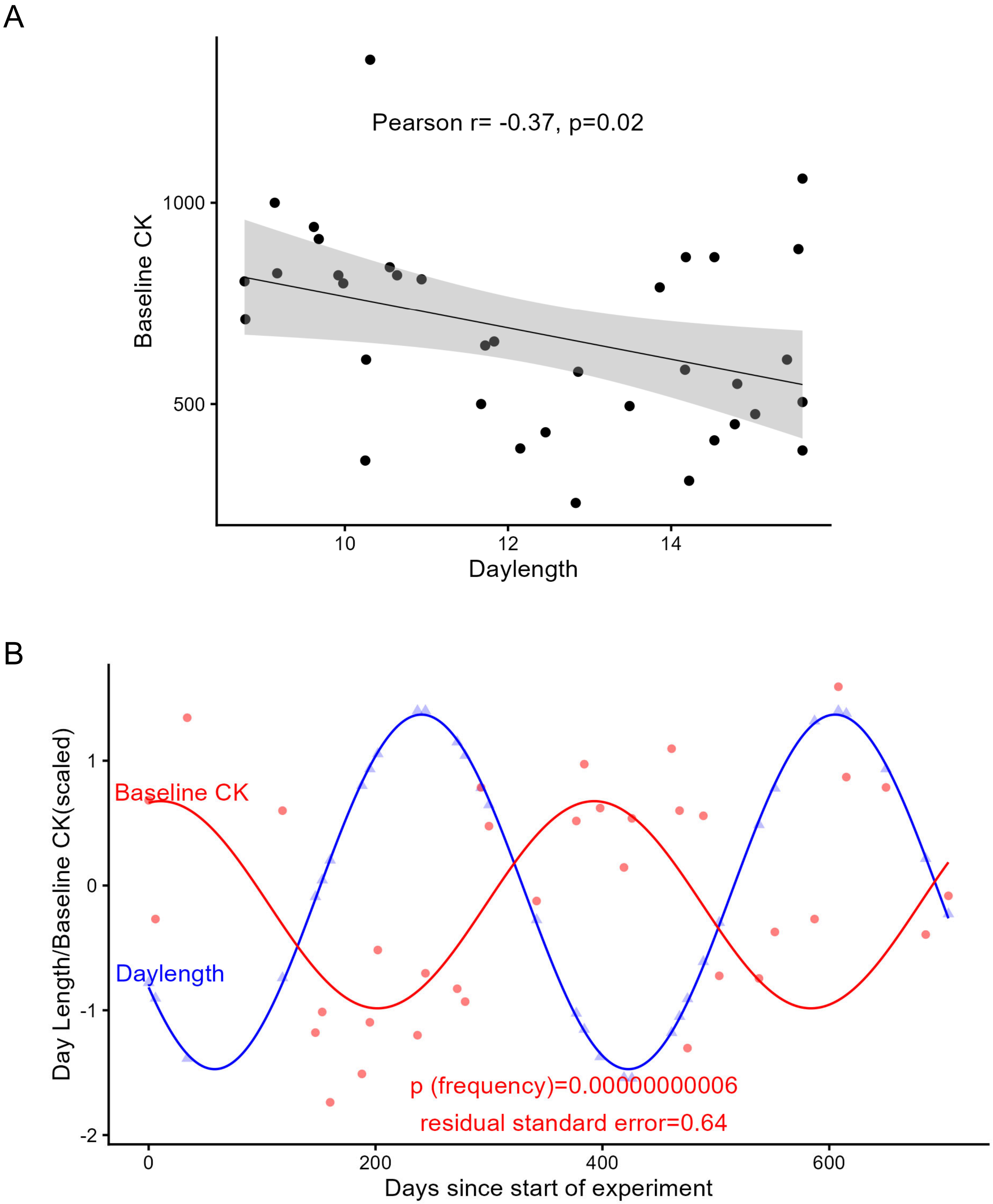

Supplementary figure 8: Variation of creatine kinase (CK) with seasonally-determined day length was significant. A) Scatter plot with Pearson r and p value.. B) Day length and baseline CK plotted with time (days since starting experiments) on the x axis. Fit sine functions are overlaid. Baseline CK varied with season and daylength, with an approximately 90-degree phase shift from day length.

Supplementary Table 1: Animals excluded for baseline abnormalities

animal	group	temp_z	map_z	plCK_kg_z	plMB_kg_z	plCR_kg_z	plK_z
39	cil	3.04	-0.47	-0.80	0.24	-0.37	-0.11
47	veh	1.68	-0.69	2.18	1.06	0.79	1.20
51	veh	1.00	2.08	-0.54	-0.70	-0.93	-0.44
53	veh	-0.43	0.13	2.08	-1.29	-0.77	-0.77
60	NI	0.20	-1.52	-0.41	-0.63	2.02	2.00
63	cil	-1.04	0.43	-0.63	3.75	0.46	-0.11
64	cil	-0.18	-1.82	0.22	-0.79	2.43	1.20
70	cil	0.50	-0.09	2.35	0.45	1.12	0.22
72	veh	0.13	-1.37	2.13	-0.42	0.66	0.87
75	cil	0.03	-0.24	0.42	-0.08	1.44	3.16
78	cil	-0.12	0.43	-0.15	-0.53	0.74	2.51
79	veh	-3.83	0.08	-1.05	-0.70	-0.17	-0.77

Highlighted values are z-scores greater than 2 or less than -2 and are the reason for exclusion.

Abbreviations: cil: cilastatin group, veh: vehicle group, NI: no impact group, temp_z: z-score for temperature, map_z: z-score for mean arterial pressure, plCK_kg_z: z-score for plasma creatine kinase per kilogram of body weight, plMB_kg_z: z-score for plasma myoglobin per kilogram of body weight, plCR_kg_z: z-score for plasma creatinine per kilogram of body weight, plK_z: z-score for plasma potassium per kilogram of body weight.

Supplementary Table 2: Animals excluded for failure to develop muscle injury

animal	group	delta_threshold	plCK_kg_delta
56	cil	35	34.04
66	veh	35	17.11
69	cil	35	28.39
76	veh	35	22.89
81	cil	35	28.67
83	cil	35	28.54

Abbreviations: cil: cilastatin group, veh: vehicle group, NI: no impact group, delta_threshold: the set threshold of plasma creatine kinase per kilogram change from baseline to 6h, below which no animal developed elevated creatinine, plCK_kg_delta: plasma creatine kinase per kilogram change from baseline to 6 hours

Supplementary Table 3: Physiologic measurements with significant correlation to day length

Measurement	Pearson	p.val
plCK	-0.375	0.024
plCl	-0.339	0.026
plHb	-0.279	0.029
plHcrit	-0.276	0.030
plBUN	-0.219	0.034
weight_kg	-0.193	0.036
tbv_ml	-0.193	0.036
plLac	-0.148	0.039
plAnGap	-0.112	0.042
etco2	-0.102	0.043

Abbreviations: plCK: plasma creatine kinase, plCl: plasma chloride, plHb: blood hemoglobin hemoglobin level, plHcrit: blood hematocrit level, plBUN: plasma urea nitrogen, weight_kg: animal weight in kilograms, tbv_mL: ventilator set tidal volume (set based on weight), plLac: plasma lactate, plAnGap: plasma anion gap, etco2: end-tidal carbon dioxide while on ventilator.
