# Supplementary material for: Efficacy of cilastatin sodium in a translational large animal crush syndrome model
Source: Commun Med (Lond). 2026 Mar 12;6:246. doi: 10.1038/s43856-026-01411-1 (PMC13121696; doi:10.1038/s43856-026-01411-1)
Supplement: Supplementary file 3 — Description of Additional Supplementary Files [file 43856_2026_1411_MOESM3_ESM.pdf]

Description of Additional Supplementary Files

Supplementary Data 1: Essential Reagents and Supplies

Supplementary Data 2-12: Extended results of statistical analysis of physiologic measurements comparing cilastatin alone with cilastatin + calcitriol (see Supplementary Results)
